# Correlation of same-visit HbA1c test with laboratory-based measurements: A MetroNet study

**DOI:** 10.1186/1471-2296-6-28

**Published:** 2005-07-13

**Authors:** Kendra L Schwartz, Joseph C Monsur, Monina G Bartoces, Patricia A West, Anne Victoria Neale

**Affiliations:** 1 Department of Family Medicine, Wayne State University School of Medicine, 101 E. Alexandrine, Detroit, MI, 48201 USA; 2 Department of Family Medicine, St. John Health System, 24911 Little Mack, St. Clair Shores, MI, 48080 USA

## Abstract

**Background:**

Glycated hemoglobin (HbA1c) results vary by analytical method. Use of same-visit HbA1c testing methodology holds the promise of more efficient patient care, and improved diabetes management. Our objective was to test the feasibility of introducing a same-visit HbA1c methodology into busy family practice centers (FPC) and to calculate the correlation between the same-visit HbA1c test and the laboratory method that the clinical site was currently using for HbA1c testing.

**Methods:**

Consecutive diabetic patients 18 years of age and older having blood samples drawn for routine laboratory analysis of HbA1c were asked to provide a capillary blood sample for same-visit testing with the BIO-RAD Micromat II. We compared the results of the same-visit test to three different laboratory methods (one FPC used two different laboratories).

**Results:**

147 paired samples were available for analysis (73 from one FPC; 74 from the other). The Pearson correlation of Micromat II and ion-exchange HPLC was 0.713 (p < 0.001). The Micromat II mean HbA1c was 6.91%, which was lower than the 7.23% from the ion-exchange HPLC analysis (p < 0.001). The correlation of Micromat II with boronate-affinity HPLC was 0.773 (p < 0.001); Micromat II mean HbA1c 6.44%, boronate-affinity HPLC mean 7.71% (p < 0.001). Correlation coefficient for Micromat II and immuno-turbidimetric analysis was 0.927 (p < 0.001); Micromat II mean HbA1c was 7.15% and mean HbA1c from the immuno-turbidimetric analysis was 7.99% (p = 0.002). Medical staff found the same-visit measurement difficult to perform due to the amount of dedicated time required for the test.

**Conclusion:**

For each of the laboratory methods, the correlation coefficient was lower than the 0.96 reported by the manufacturer. This might be due to variability introduced by the multiple users of the Micromat II machine. The mean HbA1c results were also consistently lower than those obtained from laboratory analysis. Additionally, the amount of dedicated time required to perform the assay may limit its usefulness in a busy clinical practice. Before introducing a same-visit HbA1c methodology, clinicians should compare the rapid results to their current method of analysis.

## Background

The percent HbA1c of glycated hemoglobin provides an estimate of blood glucose levels over a 3–4 month period. The HbA1c level is used for patient education and counseling, for feedback about diabetic control, to improve patient motivation, and to monitor management; thus its measurement should be optimally accurate and precise [1]. However, to date, there is no international standard for determining HbA1c [2-4], and various methodologies are commercially available. Tran [1] determined the physiological (changes over time between measurements) and analytic variation of two widely used laboratory assays, one a high performance liquid chromatography (HPLC) method, and the other an immunoassay [1]. The coefficient of variation (CV) for the HPLC was 2.6%. The 5.1% CV of the immunoassay method exceeded physiologically established limits of 2–3%, and those of the National Glycohemoglobin Standardization Program (3–4%).

Hosseini et al. [5] reported a relative ranking of assays to result in a normal HbA1c level by using the same patient's blood tested with five assays, each of which used a different method. They found that glycated hemoglobin results vary widely, with some assays consistently more likely to result in a "normal glycated hemoglobin" level than other assays, consequently resulting in differing implications for an individual patient to achieve a HbA1c level within the normal range. Ogawa [6] reported a case series where HbA1c was underestimated in the measurement by HPLC which excluded glycated abnormal hemoglobin [6]. These findings illustrate the potential usefulness for clinical practitioners to evaluate the performance of their method for determining HbA1c, especially if using different methodologies for the same patient.

Recent developments in medical technology allow clinicians to determine HbA1c test results during a patient's office visit. Several manufacturers offer an assay that can be performed by trained medical personnel and yield HbA1c results in five to ten minutes. We found only a few reports of the performance of such rapid tests used at the point of care [7,8], and one study that was conducted by the test manufacturer [9].

The objective of this pilot study was to test the feasibility of introducing a same-visit HbA1c methodology into busy family practice centers (FPCs) and to compare the results obtained from a point-of-care test with a laboratory-based technique. Specifically, our purpose was to determine: 1) if a specific rapid HbA1c methodology was accepted by medical support staff in two busy FPCs; and 2) how rapid HbA1c results compared with the standard laboratory methodology.

## Methods

### Study design

Patients were recruited for this cross-sectional study from two FPCs that are members of MetroNet, a metropolitan Detroit practice-based research network. At both sites, HbA1c analysis is routinely performed at an outside laboratory on venipuncture samples. Physicians, medical assistants, and research assistants identified consecutive diabetic patients 18 years of age and older whose physicians ordered HbA1c analysis. The study was explained to these eligible patients and informed consent obtained from those who wished to participate.

After patients were enrolled, a finger-prick blood sample was collected for in-office HbA1c testing with the BIO-RAD Micromat II. Since the BIO-RAD Micromat II is compatible with capillary, venous, and EDTA anti-coagulated blood samples, aliquots of these types were also acceptable for analysis. Research and medical staff were instructed to use finger-prick capillary samples whenever possible, but venous samples from the blood draw apparatus, or a drop of blood from the EDTA tube was substituted when necessary. At one FPC only finger-prick samples were used, while at the other FPC, thirteen MicroMat II samples were venous and five were EDTA anti-coagulated; the remaining 56 tests were performed using capillary blood samples.

The data collected included patient name, study site, the person performing same-visit HbA1c analysis, the date, and the rapid HbA1c result. Physicians were blinded to rapid HbA1c results, and relied on the laboratory analysis to make treatment decisions during the study period. One FPC used one of two different laboratories based on the patient's health insurance carrier. At one laboratory, the Primus Model 386 was used for HbA1c testing, which is a boronate – affinity HPLC method. The other laboratory used the Roche Integra 800, which uses an immuno-turbidimetric methodology. The laboratory of the second FPC used the Tosoh A1c 2.2 Plus, an ion-exchange HPLC, for analysis.

All three methodologies are aligned to Diabetes Control and Complications Trial (DCCT) and National Glycohemoglobin Standardization Program (NGSP) standards. All have linear response from HbA1c level of 3–4% to 20% or higher. The intra- and inter-assay coefficients of variation are displayed in Table 1. These values were either obtained directly from the laboratory performing the assay (Primus 386) or from the manufacturer. All are within NGSP acceptable limits.

**Table 1:** Coefficients of variation (CV) for three laboratory analyzers

**Instrument**	**Method**	**Intra-assay CV**	**Inter-assay CV**
Primus 386	Boronate affinity HPLC	0.9%	2.9%
Roche Integra 800	Immuno-turbidimetric	2.3%	2.4%
Tosoh A1c2.2 Plus	Ion-exchange HPLC	1.3%	< 4.0%

The BIO-RAD Micromat II, which provides results in approximately 5 minutes, incorporates an affinity chromatography method that measures the percent glycated hemoglobin in the sample. According to the manufacturer, the analyzer then uses a factory-set algorithm to deliver an HbA1c result which is calibrated to the recommendations of the DCCT and is traceable to the NGSP. The intra-assay coefficient of variation is reported to range from 2.93 – 4.65%; higher at lower values of HbA1c. The inter-assay coefficient of variation is estimated to be higher; however values are not given in the package insert. The sensitivity of the assay ranges from 4 – 15% HbA1c. BIO-RAD representatives provided an in-service to help familiarize staff in the use and operation of the Micromat II analyzer.

Each HbA1c analysis with the Micromat II requires a single test cartridge, which consists of several tubes with reagents that are mixed and decanted into a collection reservoir for measurement. After a test cartridge has been placed into the Micromat II, a 20 microliter blood sample is added to the first tube. This initiates a series of aliquot additions and incubation steps. In total there are four decanting steps followed by four incubations. These incubations require a total time of 230 seconds and range from 40 seconds to 80 seconds in length. Quality control procedures were carried out as outlined in the Micromat II instruction manual. Controls and standards were run per the manufacturer's recommendation; results were always acceptable.

### Analytic strategy

Data were analyzed separately by type of laboratory methodology. To evaluate the performance of the BIO-RAD Micromat II, Pearson correlations were calculated using the laboratory results as the standard. Scatter plots and regression lines were also examined. The mean absolute difference between the sample groups was determined to test the hypothesis that group means are equal (α = 0.05), using a two-sided paired t-test.

## Results

One hundred fifty-six patients were enrolled into the study (75 from one FPC, and 81 from the other FPC). Nine different medical staff performed the rapid HbA1c testing. Data from nine patients were omitted: eight had missing laboratory results, and one result was out of the precision range of the machine (HbA1c = 18.1%). Therefore, 147 paired samples were available for analysis, 73 from one site and 74 from the other.

**Figure 1:**
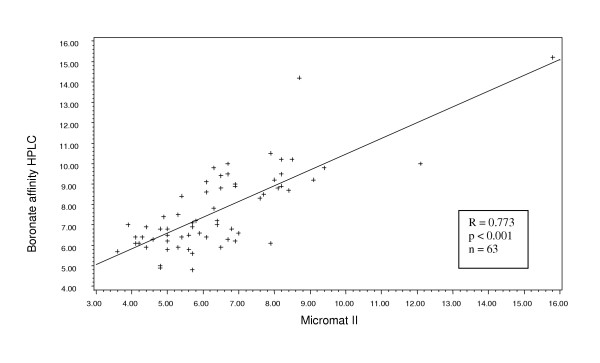
Scatterplot and regression line of HbA1c values produced from boronate affinity HPLC and Micromat II.

**Figure 2:**
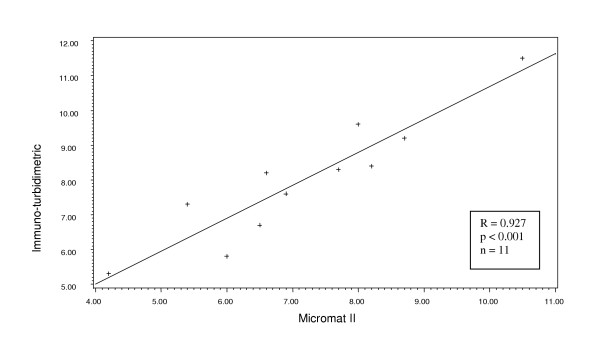
Scatterplot and regression line of HbA1c values produced from immuno-turbidimetric and Micromat II methods.

**Figure 3:**
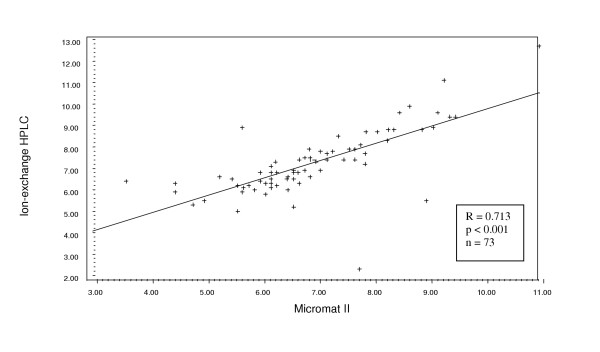
Scatterplot and regression line of HbA1c values produced from ion-exchange HPLC and Micromat II methods.

Considering first the data from the site that used two different laboratories: the boronate-affinity HPLC (n = 63) and the immuno-turbidimetric (n = 11), we found a significant correlation with the Micromat II results for both (Pearson r = 0.773, p < 0.001 and r = 0.927, p < 0.001, respectively) (Figures 1 and 2). The range of values was from 2.3% to 12.70%. The laboratory method yielded a mean HbA1c value that was significantly higher than that from the Micromat II for both methodologies (7.71 ± 1.99 vs. 6.44 ± 1.99, p < 0.001 and 7.99 ± 1.76 vs. 7.15 ± 1.72, p = 0.002, respectively (Table 1). Similarly, the Micromat II correlated well with the ion-exchange HPLC (n = 73, Pearson r = 0.713, p < 0.001) (Figure 3). Again, the mean HbA1c result from the laboratory was significantly greater than the mean from the Micromat II (7.23 ± 1.51 vs. 6.91 ± 1.34, p = 0.014). The range of results from these two methods was 3.6% to 15.80%.

Regarding feasibility and acceptability of introducing the same-visit Micromat II test into the busy clinical practice setting, we found that medical assistants were able to collect and analyze samples and produce same-visit results. However, the five minute time dedication for each individual analysis was not well tolerated by staff because of numerous competing demands that made it difficult to perform all the test steps in the time intervals prescribed.

## Discussion

Physicians in ambulatory settings routinely send blood samples to laboratories for HbA1c testing, and then wait several days for the HbA1c test results. Thus, patient counseling and treatment adjustments based on HbA1c levels are delayed, and at times follow-up can be lost completely.

Recent advancements in technology now make it possible for physicians to incorporate point-of-care HbA1c results to evaluate and adjust treatment of their diabetic patients. Studies of the effect of same-visit HbA1c measurement found significantly improved glycemic control through 12-month follow-up [10,11]. This technology is gaining acceptance, and is now offered by a number of manufacturers. The same-visit HbA1c test provides the opportunity to improve diabetes care by discussing the value and adjusting management as needed during the same-visit, rather than waiting until the patient can be telephoned and/or scheduled for a future visit. HbA1c testing has been studied for its effect on improved glycemic control in trials primarily conducted in specialty clinics. Yet, little is published regarding the validity of the same-visit test result, and the feasibility of using a same-visit methodology in a busy primary care setting.

The manufacturer reports a correlation coefficient of 0.96 between the BIO-RAD Micromat II and HPLC methodology. However, the correlation coefficients we obtained in this clinical situation (r = 0.713; and r = 0.773 for the two different HPLC methods) were less than reported by the company. The highest correlation was with the immuno-turbidimetric methodology (r = 0.927). The mean HbA1c level obtained from Micromat II was significantly lower than that yielded from the three types of laboratory analysis, and this difference spanned the treatment threshold level currently recommended by the American Diabetes Association (ADA) [12]. Thus, for some patients, the Micromat II rapid test yielded a test result that was below the ADA treatment threshold of 7%, while the laboratory analysis produced a test result above 7%, suggesting the need for more intensive therapy.

### Limitations

There are likely limitations to the generalizability of the study findings. First, the number of medical staff (n = 9) that collected samples and performed the HbA1c testing may have increased the variability of the same-visit test results. Similarly, the correlation between the laboratory and the same-visit methodologies may be improved when conducted under ideal conditions where sources of variation in the operation of the Micromat II are minimized. Secondly, introducing a research study into a busy clinical practice setting is often met with varying degrees of resistance. Thus, evaluating the acceptance of what may have been viewed by staff as a research technique may have limitations when generalizing the acceptance of a clinical procedure. However, our purpose was to conduct a correlation study in the real world setting of the busy FPC. We trained all clinical staff in the calibration and specimen analysis of the point-of-care instrument. From discussions with the clinical staff and physicians, we learned that there was variability among staff members to faithfully adhere to the Micromat II timed steps as outlined in the test kit instructions.

## Conclusion

Same-visit HbA1c testing offers potential benefits for diabetes care, as patient results are available in the same-visit. However, clinicians should be aware that the rapid HbA1c technology may produce results that are lower than the method that they have been utilizing, and that the same-visit test may suggest a different treatment strategy than a result from their usual laboratory testing source. To overcome this barrier, we suggest that clinicians determine how the results of a same-visit HbA1c test compare with the outside laboratory reports on which they routinely base their treatment plans before incorporating the same-visit HbA1c test into their practice.

## Abbreviations

HbA1c – Hemoglobin A1c

## Competing interests

The author(s) declare that they have no competing interests.

## Authors' contributions

KS, JM and AVN developed the idea. KS, PW, and AVN designed and oversaw data collection. KS and AVN supervised statistical analyses performed by JM and MB. All authors contributed to the interpretation of the data and the writing and editing of the manuscript.

**Table 2:** Comparison of mean percent HbA1c (SD) of paired samples using three laboratory methodologies and BIO-RAD Micromat II same-visit assay

	**Mean percent HbA1c (SD)**		
**Laboratory Methodology**	**HPLC**	**Micromat II**	**p-value**

Boronate affinity HPLC (n = 63)	7.71 (1.99)	6.44 (1.99)	<0.001
Immuno-tubidimetric (n = 11)	7.99 (1.76)	7.15 (1.72)	0.002
Ion-exchange HPLC (n = 73)	7.23 (1.51)	6.91 (1.34)	0.014

## Pre-publication history

The pre-publication history for this paper can be accessed here:


